# Klaus Bergmann, MD, FRCPsych

**DOI:** 10.1192/bjb.2021.11

**Published:** 2021-08

**Authors:** Robin Jacoby, Robert Howard

Formerly Consultant Old Age Psychiatrist at the Bethlem Royal and Maudsley Joint Hospital, London, UK

**Figure:**
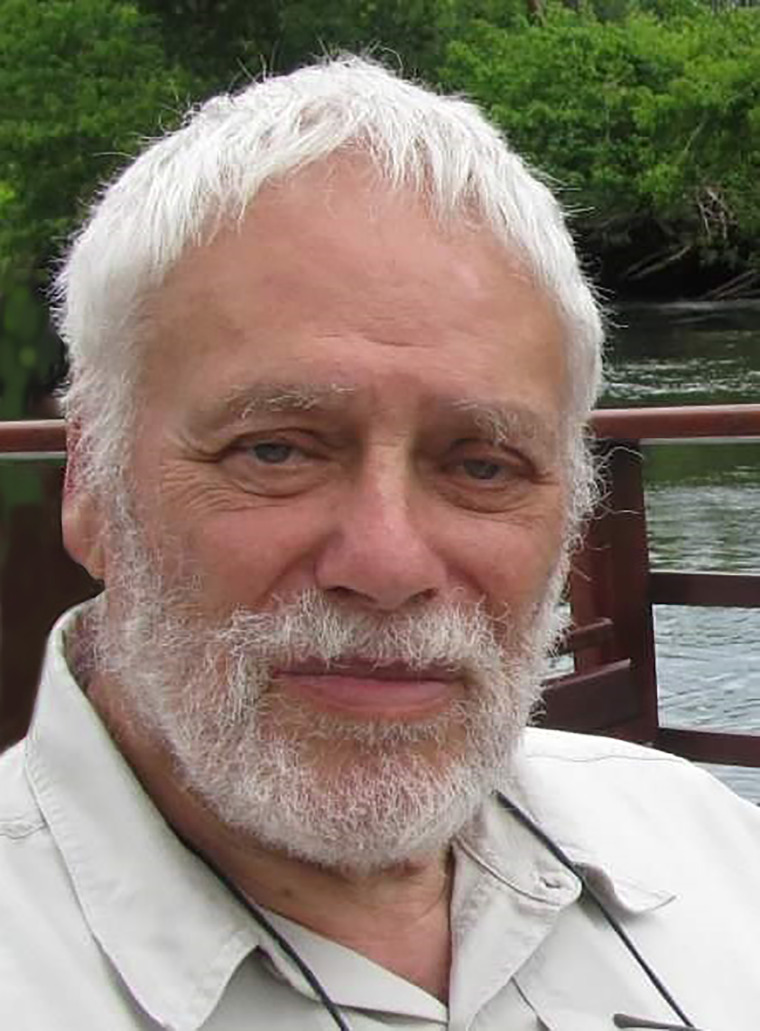

Klaus Bergmann, who died following a stroke on 5 December 2020, was one of the towering figures in British old age psychiatry in its early years, playing a major role in bringing the specialty to maturity. In the 1960s and 1970s, he was based in the Department of Psychiatry at Newcastle University, headed by Martin Roth. Mentored by David Kay and in collaboration with Gary Blessed and Bernard Tomlinson, Klaus was deeply involved in one of the first community cohort studies of the prevalence and outcomes of old age mental disorders outside hospitals. The important findings of these studies have dictated policy ever since.^1,2^

Although involved in research, Klaus was a superb clinician who cared more about his patients than he did about anything else at his work. His acumen in diagnosis and treatment was second to none. He was the reason many of his trainees went into old age psychiatry, some of those now occupying the highest levels in the profession.

He was born in 1930 in Dresden, younger brother to two older sisters, to Fritz and Alice Bergmann. Fritz owned a millinery firm that he was able to re-establish in Luton after emigration from Germany. In 1937, before the Second World War, his Jewish family moved via Palestine to England. He would often recount his childhood disappointment that he had not been allowed to wear the uniform and carry the dagger of the Hitler Youth, which he coveted.

Klaus was educated first at St George's School, Harpenden, following which he studied medicine at Sheffield University, where he met his wife Marie, a fellow student reading German. After National Service, he returned to Sheffield for psychiatric training under Erwin Stengel, also a refugee from the Nazis. This gave him a broad outlook based both on rigorous history-taking and mental state examination, but with psychoanalytic insights. He always maintained an affectionate respect for Stengel but, as was the case with others among his senior colleagues, he could never resist telling humorous stories about them. This characteristic betrayed both immense respect for those Klaus deemed worthy, combined with a refusal to adopt blinkered hero worship.

In 1964, Klaus moved from Sheffield to Newcastle, where the Department of Psychiatry was headed by Martin Roth, and it was here that he entered old age psychiatry. Klaus often said that he regarded Roth as the Kraepelin of old age psychiatry, i.e. a founding father in terms of classification and phenomenology. Once again, Klaus enjoyed poking fun at him without ever losing respect for his important contributions to the specialty. In 1966, Klaus took up a consultant post at St Francis Hospital, Haywards Heath, Sussex, where he was overworked and grossly under-resourced. So, in 1969, he returned to Newcastle, continuing to carry out research in the community.

At heart, Klaus was a clinician and a teacher and, in 1979, at the invitation of Raymond Levy, he moved down to London to the Bethlem Royal and Maudsley Hospital as a consultant old age psychiatrist, replacing Felix Post, who had retired. With typically wry humour he asked Raymond: ‘Do you think they'll mind that I'm not a gentleman?’. As a matter of fact, they did not mind, and he settled in there very well. At the Maudsley he worked alongside Raymond Levy, who became Professor of Old Age Psychiatry. Later, they were joined by Robin Jacoby and Marisa Silverman (now Parrish).

Klaus inspired loyalty through his clinical skills and his courage. He was not frightened to offend people in defence of his patients. For example, if he saw something that he regarded as shocking, he would, himself, shock the perpetrator. One day, he arrived at a care home to be led into the day-room, where all the residents were sitting round the walls except for one old lady who was on a commode in the middle of the room, incompletely shielded by one or two movable screens. Klaus turned to the care-home manager and said: ‘Oh, do you sh** in your sitting-room?’.

He was often incredibly funny. Never completely shaking off his adolescent schoolboy personality, he was a fount of good and very non-PC jokes, designed on occasions to shock some of the more prudish people at the Maudsley. His office was a chaotic mess, with journals and committee papers all over the desk and the floor. His colleagues used to joke that he would not be able to retire but would have to be removed as a hoarder under a section of the Public Health Act. This delighted him, and he often boasted about it. In the end, he retired without recourse to the Public Health Act. Early on in his retirement he wrote reports for the General Nursing Council in connection with disciplinary action. He was rather fond of what he called his ‘naughty nurses’.

Klaus enjoyed a long and happy marriage to Marie, who survives him with their son George, their daughter Caroline, and three grandchildren. Vicky, their elder daughter, died of cancer in 2014.
